# Gray Matter Differences in Adolescent Psychiatric Inpatients: A Machine Learning Study of Bipolar Disorder and Other Psychopathologies

**DOI:** 10.1002/brb3.70589

**Published:** 2025-06-10

**Authors:** Renata Rozovsky, Maria Wolfe, Halimah Abdul‐waalee, Mariah Chobany, Greeshma Malgireddy, Jonathan A. Hart, Brianna Lepore, Farzan Vahedifard, Mary L. Phillips, Boris Birmaher, Alex Skeba, Rasim S. Diler, Michele A. Bertocci

**Affiliations:** ^1^ Department of Psychiatry University of Pittsburgh Pittsburgh Pennsylvania USA; ^2^ Western Psychiatric Hospital University of Pittsburgh Medical Center (UPMC) Pittsburgh Pennsylvania USA

## Abstract

**Background:**

Bipolar disorder (BD) is among the psychiatric disorders most prone to misdiagnosis, with both false positives and false negatives resulting in treatment delay. We employed a whole‐brain machine learning approach focusing on gray matter volumes (GMVs) to contribute to defining objective biomarkers of BD and discriminating it from other forms of psychopathology, including subthreshold manic presentations without a BD Type I/II diagnosis.

**Methods:**

Five support vector machine (SVM) models were used to detect differences in GMVs between inpatient adolescents aged 13–17 with BD‐I/II (*n* = 34), other specified BD (OSB) (*n* = 106), other non‐bipolar psychopathology (OP) (*n* = 52), and healthy controls (HC) (*n* = 27). We examined the most discriminative GMVs and tested their associations with clinical symptoms.

**Results:**

Whole‐brain classifiers in the model BD‐I/II versus OSB achieved total accuracy of 79%, (AUC = 0.70, *p* = 0.002); BD versus OP 66%, (AUC = 0.61, *p* = 0.014); BD versus HC 66%, (AUC = 0.67, *p* = 0.011); OSB versus HC 77%, (AUC = 0.61, *p* = 0.01); OP versus HC 68%, (AUC = 0.70, *p* = 0.001). The most discriminative GMVs that contributed to the classification were in areas associated with movement, sensory processing, and cognitive control. Correlations between these GMVs and self‐reported mania, negative affect, or anxiety were observed in all inpatient groups.

**Conclusions:**

These findings indicate that pattern recognition models focusing on GMVs in regions associated with movement, sensory processing, and cognitive control can effectively distinguish well‐characterized BD‐I/II from other forms of psychopathology, including other specified BD, in a pediatric population. These results may contribute to enhancing diagnostic accuracy and guiding earlier, more targeted interventions.

## Graphical abstract

We used a whole‐brain machine learning approach to investigate gray matter volumes in 219 adolescent inpatients with bipolar disorder (BD‐I/II, OSB), other psychopathologies, or healthy controls. Classification accuracies reached up to 79%, with key regions in movement, sensory, and cognitive networks driving the strongest differentiation. These findings highlight potential neurobiological biomarkers for enhancing early diagnostic accuracy and guiding targeted interventions for pediatric BD.

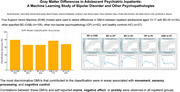

## 1 Introduction

Bipolar disorder (BD) is one of the most severe psychiatric conditions, affecting 1%–3% of the adult population worldwide. BD commonly manifests during adolescence (Kowatch et al. 2005; Kowatch et al. 2005; Pavuluri et al. 2005); however, delays in accurate diagnosis often result in delayed appropriate treatments (Bolge et al. 2008; Bowden 2005; Leverich et al. 2007) and lead to impaired psychosocial functioning, substance abuse, and suicide (Chang et al. 2006; Leibenluft and Rich 2008; Leverich et al. 2002; Leverich et al. 2003; Merikangas et al. 2007; Perlis et al. 2004), underscoring the importance of early identification and treatment as key research priorities in BD (Duffy et al. 2017). However, early identification is often hindered by the high comorbidity of attention deficit/hyperactivity disorder (ADHD), disruptive behavior disorders, and anxiety disorders, as well as by overlapping clinical features—subthreshold mania/hypomania, ADHD‐like symptoms, and major depressive disorder (MDD)‐related presentations— that are difficult to disentangle (Arnold et al. 2011; Birmaher et al. 2009; Leibenluft et al. 2003; Lewinsohn et al. 1995). Identifying objective biomarkers reflecting underlying pathophysiological processes, as well as detecting BD‐specific symptoms associated with these biomarkers, is therefore crucial to improving the differentiation of BD from other psychiatric conditions.

Although previous studies have reported structural abnormalities in BD (including parietal, frontal, and subcortical regions) (Chang et al. 2005; Cui et al. 2020; Frazier, Ahn, et al. 2005; Frazier, Breeze, et al. 2005; Gao et al. 2013; Gao et al. 2021; Johnston et al. 2017; Keramatian et al. 2021; MacMaster et al. 2014; Sandoval et al. 2016; Saxena et al. 2023; Xiao et al. 2020; Y. Zhang et al. 2021), few have attempted to use a data‐driven, whole‐brain machine learning classification specifically in adolescents, and none have directly contrasted BD to individuals with “other specified BD” (OSB)— a population at high risk for converting to BD‐I or BD‐II with subthreshold manic/hypomanic symptoms. The importance of distinguishing between BD‐I/II and OSB is clinically relevant because only a subset of them will develop full‐blown BD and require long‐term focused treatment, OSB presentation conversion rates to BD‐I/II are approximately 30% in 2 years and 50% in 4 years (Birmaher et al. 2009). The ongoing Inpatient Child and Adolescent Bipolar Spectrum Imaging study (InCabs Imaging) is uniquely designed to identify structural differences in at‐risk and disease states. This study integrates multi‐day and multi‐observer observation and assessment during hospitalization to classify patients into three groups: (Kowatch et al. 2005) well‐characterized bipolar I/II disorder (BD‐I/II); (Kowatch et al. 2005) OSB; and (Pavuluri et al. 2005) other psychopathologies (OP), importantly including non‐bipolar depressive disorders, ADHD, anxiety disorders, and disruptive behavior disorders.

Gray matter volumes (GMV) were selected for this analysis as it is relatively stable and reliable over time (Jovicich et al. 2013; Madan and Kensinger 2017; Melzer et al. 2020). Studies have shown that relative to healthy youth, pediatric BD is associated with altered patterns of GMV in cortical regions involved in movement, sensory processing, and sensory sensitivity, including the superior temporal gyrus (STG), inferior frontal gyrus (IFG), precentral gyrus, inferior parietal lobule, and cerebellar regions (Saxena et al. 2023; Xiao et al. 2020; Y. Zhang et al. 2021; Dou et al. 2022). Subcortical regions functionally associated with movement, emotion, and memory, including the putamen, amygdala, and hippocampus, also distinguished BD youth from healthy controls (Cui et al. 2020; Gao et al. 2021; Xiao et al. 2020; Dou et al. 2022). There were no differences observed comparing BD subtypes and between BD and unipolar depression in small regions of interest studies (Xiao et al. 2020; Y. Zhang et al. 2021) but sample sizes were small and advances in machine learning may improve detection.

A machine learning approach, specifically support vector machines (SVMs), was selected here due to its robustness in classification tasks. It is a supervised machine learning approach that has emerged as a promising technique for discovering neuroimaging‐based biomarkers of psychiatric disorders in adult populations (Mateos‐Pérez et al. 2018; Wolfers et al. 2015; Ecker et al. 2010; Gong et al. 2011; Zhou et al. 2018; Achalia et al. 2020; Bayes et al. 2021; Colombo et al. 2022; Jan et al. 2021). Over the past decade, SVM classifications have been successfully applied to structural MRI in adult populations aiming to: distinguish healthy individuals from patients with a variety of psychiatric disorders; predict diagnoses; or differentiate among patients with diverse psychiatric conditions. The SVM algorithm establishes a model by identifying complex patterns in the high dimensional data that are linearly separable, maximizes the margins between the hyperplanes, and minimizes overfitting in moderate sample sizes (Noble 2006).

However, to our knowledge, only one study has focused on using a machine learning approach to differentiate GMV patterns between healthy participants and BD in the pediatric populations (Dou et al. 2022), and no prior study has used machine learning‐based pattern recognition approaches to distinguish BD from OSB and from other severe psychopathologies in an inpatient pediatric sample. Therefore, using an SVM classification with whole‐brain GMV data in a carefully characterized adolescent inpatient cohort, we aimed: (1) To differentiate BD‐I/II from OP, including both OSB and OP, as well as from HC, and (2) To identify the GMVs that most contributed to the classification models and examine whether these GMVs were associated with specific self‐reported clinical symptoms.

We hypothesized that:

1. Whole‐brain GMV SVM classification would show good accuracy in distinguishing BD‐I/II from healthy participants and from individuals with other forms of psychopathology, (OSB and OP).
2. The specific GMVs with the highest weights would correlate with mania or related clinical symptoms, consistent with prior structural findings in mood disorders.

If supported, these findings could contribute to the development of a risk calculator for BD, improving diagnostic accuracy in pediatric populations.

## 2 Methods

### 2.1 Participants

Two hundred twenty‐six adolescents were recruited from the ongoing InCabs study (R01 MH‐121451, “From Manic Symptoms to Bipolar: Neural Behavioral Markers Using Two Analytical Models”). After the exclusion of seven participants due to missing data (*n* = 5), artifacts (*n* = 1), and brain pathology (*n* = 1), the final sample included two hundred nineteen participants: inpatients with BD‐I/II (*n* = 34, 27 females), OP (*n* = 52, 36 females), OSB (*n* = 106, 82 females), and HC (*n* = 27, 14 females). Within our small age range, participants differed in age with BD‐I/II participants older and HC younger than the other groups (*p* < 0.04) and a greater proportion of females in the inpatient samples (*p* < 0.04). Groups did not differ on race or Hispanic origins (See Table 1 demographics and clinical measures). All inpatient participants were receiving pharmacological treatment. Exclusion criteria included: history of serious medical illness, head injury, or neurological disorder; IQ < 70, assessed with Wechsler Abbreviated Scale of Intelligence (Wechsler 1999); diagnosis of autism, eating disorder, schizophrenia, or severe substance use disorder, and magnetic resonance imaging (MRI) contraindication (e.g., pregnancy, metal in the body). This study was approved by the Human Research Protection Office at the University of Pittsburgh. Participants received monetary compensation. Parents/guardians provided informed consent, and adolescents provided informed assent prior to participation.

**TABLE 1:** Demographic (a) and clinical (b) measures across BD‐I/II, OP, OSB, and HC groups.

a.		**BD = 34**	**OP = 52**	**OSB = 106**	**HC = 27**		
Age mean (SD)		15.53 (1.1)	15.23 (1.2)	14.96 (1.3)	14.67 (1.6)	*F* = 2.72	*p* = 0.04
Gender (F)		27	36	82	14	x2 = 8.14	*p *= 0.04
Race		—	—			x2 = 11.44	*p* = 0.08
	Black	2	17	23	7	—	
	White	32	33	80	20	—	
	Other*	0	2	3	0	—	
Hispanic (yes)		3	1	1	2	x2=7.04	*p* = 0.07
*Other race includes Asian and American Indian/Alaska Native, and Native Hawaiian.							
b.							
BCMRS mean (SD)	Month	12.70 (5.5)	8.23 (4.05)	11.90 (4.9)		*F = *9.404	*p* < 0.001
	Lifetime	15.19 (5.2)	8.63 (3.6)	12.44 (Leverich et al. 2007)		*F = *13.742	*p* = < 0.001
SCARED mean (SD)		46.73 (18.84)	37.74 (19.9)	43.61 (18.32)		*F = *2.193	*p* = 0.116
PANAS mean (SD)	Positive affect	39.47 (Perlis et al. 2004)	36.12 (12.42)	18.38 (12.04)		*F = 0*.766	*p* = 0.467

	Negative affect	49.43 (15.13)	43.30 (14.62)	48.88 (13.69)		*F = *2.434	*p* = 0.091
MFQ mean (SD)		44.06 (15.89)	36.45 (16.15)	42.23 (15.15)		*F = *2.517	*p* = 0.084


*Note*: All clinical measures are self‐reported by the participant. The subset of participants completed self‐report measures.

Abbreviations: BCMRS = Brief Child Mania Rating Scale; BD = bipolar disorder group; OSB = other specified bipolar disorder group; HC = healthy controls; MFQ = Mood and Feelings Questionnaire; OP = other psychopathology group; PANAS = Positive and Negative Affect Scale; SCARED = Screen for Child Anxiety Related Disorders.

### 2.2 Clinical Assessment

All clinical diagnostic staff was directly trained by, and all psychiatric diagnoses were confirmed through consensus meetings by Rasim Diler, MD, a licensed, board‐certified, pediatric psychiatrist specializing in pediatric BD and director of the Inpatient Child and Adolescent Bipolar Services (InCabs) unit at Western Psychiatric Hospital. DSM 5 (Regier et al. 2013) criteria including sustained periods of elevated mood or energy are outlined in the DSM 5 and were used for final BD‐I/II and OP diagnoses. OSB was defined as the presence of a brief (4 h/day and 4‐life time) cluster of DSM manic symptoms that caused impairment as described before (Axelson et al. 2006). All instruments had good psychometric properties and included the Kiddie Schedule for Affective Disorders and Schizophrenia for School‐Age Children (K‐SADS) (Kaufman et al. 1997), the Brief Child Mania Rating Scale (BCMRS) (Henry et al. 2008; Pavuluri et al. 2006) to measure mania in the past month and lifetime, the Screen for Child Anxiety Related Disorders (SCARED) to measure anxiety (Behrens et al. 2019; Birmaher et al. 1999), the Mood and Feelings Questionnaire (MFQ) to measure depression (Sund et al. 2001; Wood et al. 1995), and the Positive and Negative Affect Scale (PANAS) (Laurent et al. 1999) to measure positive affect and negative affect. Inpatient groups differed on self‐reported lifetime and past month mania ratings (ps<.001), but did not differ on self‐reported ratings of anxiety, positive affect, negative affect, or depression (all *ps* > 0.084) (Table 1).

### 2.3 Neuroimaging Data Acquisition

Structural neuroimaging data were collected at the University of Pittsburgh using a 3.0 Tesla Siemens Magnetom Prisma MRI scanner. Structural sagittal MPRAGE images were acquired with the following parameters: TR = 2300 ms, TE = 2.9 ms, flip angle = 9°, FOV = 256 × 256 mm, voxel size = 1 mm isotropic, with 176 continuous slices.

### 2.4 Neuroimaging Data Processing

Structural MRI images were preprocessed using the statistical parametric mapping software package (SPM12, http://www.fil.ion.ucl.ac.uk/spm) running on Matlab 2023a (MathWorks, Natick, MA, USA). All scans underwent a procedure similar to Unified segmentation (Ashburner and Friston 2005), including segmentation, bias correction, and spatial normalization. GM images were then smoothed with a 12‐mm FWHM Gaussian kernel for further analysis. Although smoothing leads to some loss of spatial information, 12 mm smoothing effectively increases the signal‐to‐noise ratio and compensates for residual anatomical variability after spatial normalization, thereby enhancing the sensitivity of machine learning classification (Merhof et al. 2011; Matsuda et al. 2002).

### 2.5 Machine Learning Analysis

#### 2.5.1 Classification

Classification was performed using the Pattern Recognition for Neuroimaging Toolbox (PRoNTo) (http://www.mlnl.cs.ucl.ac.uk/pronto (Schrouff et al. 2013), employing a supervised SVM with a kernel (‐t 4) to handle the high dimensionality of gray matter data (Wolfers et al. 2015). Each preprocessed gray matter image was treated as a high‐dimensional data point, with each voxel representing a feature. Linear decision boundaries then classified scans based on their class label. The SVM then identifies the maximum‐margin decision boundary (hyperplane) that best separates the classes (Ojala and Garriga 2009).

Nested 10‐ to 14‐fold cross‐validation (CV) was employed to estimate the optimal cost parameter (C) from the set [0.01, 0.1, 1, 10, 100, 1000] and to assess the model's generalizability (Orrù et al. 2012; Schrouff et al. 2018). We selected the number of folds for each model to ensure that each fold was representative of the overall sample and maintained a balanced representation across groups, thereby improving the reliability of performance metrics. No single aggregated C value was chosen globally; instead, each outer fold had its own inner validation loop, which could yield different best values of C, ensuring that hyperparameter tuning remains strictly within each fold's training data to prevent overfitting. After identification of the optimal C in each outer fold, the model was trained and tested on a k‐fold CV approach on subjects per group. In this scheme, each group's subjects were partitioned into k folds, and in each run, one fold from every group was held out as the test set while the remaining folds formed the training set. This procedure ensured that every test set was representative of all groups, providing an estimate of the classifier's generalizability (Orrù et al. 2012; Schnyer et al. 2017).

**FIGURE 1:**
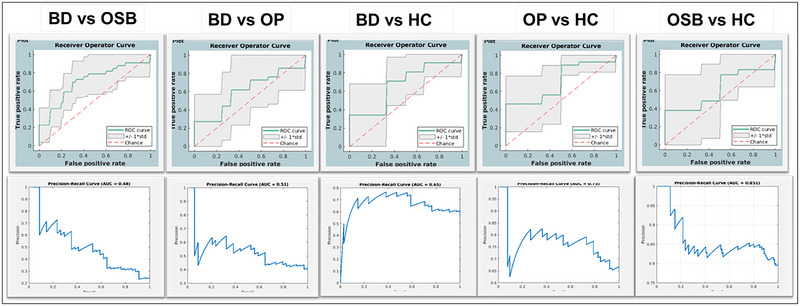
**Classification performance**. Panel **(A)** shows the receiver operator characteristic (ROC) curves with shaded regions indicating ±1 standard deviation. The diagonal dashed line represents chance level performance. Panel **(B)** illustrates corresponding precision–recall curves with respective area under the curve (AUC) scores provided at the top of each subplot.

To evaluate classifier performance, the receiver operating characteristic (ROC) curve and the area under the curve (AUC) were calculated (Figure 1). The ROC curve, derived from probabilistic classifications, compares the classifier's true positive and false positive rates as the decision threshold varies (Fawcett 2006; Mahmoudi et al. 2012). AUC represents the discriminative power of a classifier, with perfect classification achieving an AUC of 1 (Lim et al. 2013). A 1000‐permutation test (Schrouff et al. 2013; Ojala and Garriga 2009) assessed the statistical significance of accuracy, AUC, and other metrics. In addition, precision and recall were calculated for each model (Table 2) (for Precision‐Recall (PR) and ROC curves, see Figure 1). Because class imbalance can inflate overall accuracy, we also computed balanced accuracy and predictive values for each class. To further explore class imbalance, we conducted a sensitivity analysis using random undersampling for the BD versus OSB and OSB versus HC comparisons (detailed below).

**TABLE 2:** Results of classification models performance, each had 1000 permutations. Statistics and the top 15 brain GMVs ranked by percentage of contribution to the model performance.

BD‐I/II vs. OSB. Class 1: BD, *n* = 34; Class 2: OSB, *n* = 106		
Total accuracy, %	79	—
Balanced accuracy, %	64	*p* = 0.008
Class 1 predictive value, %	67	—
Class 2 predictive value, %	82	—
AUC	0.7	*p* = 0.002
Precision class 1/class 2	0.63/0.82	—
Recall class 1/class 2	0.35/0.93	—
**Region**	**Weights %**	**Voxels**
Left cerebellum 10	3.06	117
Right cerebellum 10	2.39	113
Right cerebellum 7b	1.81	680
Left cerebellum 9	1.58	1184
Right postcentral gyrus	1.54	6549
Right superior parietal gyrus	1.53	2733
Left superior parietal gyrus	1.52	3295
Left caudate	1.42	1462
Left transverse temporal (Heschl's) gyrus	1.4	538
Right caudate	1.31	1555
Left paracentral lobule	1.3	1905
Right precentral gyrus	1.27	5952
Left angular gyrus	1.2	2541
Right cerebellum 8	1.2	2403
Right putamen	1.19	2117
**BD‐I/II vs. OP, Class 1: BD, *n* = 34, Class 2: OP, *n* = 52**		
Total accuracy, %	66	—
Balanced accuracy, %	64	*p* = 0.029
Class 1 predictive value, %	54	—
Class 2 predictive value, %	75	—
AUC	0.61	*p* = 0.014
Precision class 1/class 2	0.57/0.62	—
Recall class 1/class 2	0.5/0.59	—
**Region**	**Weights %**	**Voxels**
Left paracentral lobule	2.6	1905
Left transverse temporal (Heschl's) gyrus	1.71	538
Right cerebellum 7b	1.45	680
Right cerebellum 3	1.45	393
Left middle temporal pole	1.4	1212
Right cerebellum 8	1.38	2403
Left cerebellum 7b	1.34	781
Right cerebellum 10	1.33	113
Left cerebellum 10	1.31	117
Right 0aracentral lobule	1.29	1457
Right inferior frontal operculum	1.25	2490
Right caudate	1.24	1555
Left pallidum	1.2	445
Left cerebellum 9	1.17	1184
Right superior parietal gyrus	1.16	2733
**BD‐I/II vs. HC, Class 1: BD, *n* = 34, Class 2: HC, *n* = 27**		
Total accuracy, %	66	—
Balanced accuracy, %	65	*p* = 0.04
Class 1 predictive value, %	69	—
Class 2 predictive value, %	67	—
AUC	0.67	*p* = 0.011
Precision class 1/class 2	0.69/0.62	—
Recall class 1/class 2	0.71/0.59	—
**Region**	**Weights %**	**Voxels**
Left cerebellum 10	1.85	117
Left paracentral lobule	1.59	1905
Right cerebellum 10	1.57	113
Left inferior frontal operculum	1.57	1991
Left inferior frontal triangularus	1.42	4360
Right inferior parietal gyrus	1.37	2476
Left superior parietal gyrus	1.35	3295
Vermis 3	1.35	448
Left angular	1.34	2541
Right middle occipital gyrus	1.33	4359
Left pallidum	1.33	445
Left middle frontal gyrus	1.23	8279
Left supplemental motor area	1.22	4180
Left cerebellum 9	1.2	1184
Left cerebellum crus 1	1.2	5186
**OP vs. HC, Class 1: OP, *n* = 52, Class 2: HC, *n* = 27**		
Total accuracy, %	68	—
Balanced accuracy, %	63	*p* = 0.039
Class 1 predictive value, %	75	—
Class 2 predictive value, %	53	—
AUC	0.7	*p* = 0.001
Precision class 1/class 2	0.73/0.52	—
Recall class 1/class 2	0.79/0.44	—
**Region**	**Weights %**	**Voxels**
Right paracentral lobule	1.89	1457
Left middle temporal pole	1.66	1212
Left paracentral lobule	1.64	1905
Left precuneus	1.53	6956
Right precentral gyrus	1.42	5952
Vermis 3	1.41	448
Left inferior frontal operculum	1.3	1991
Right inferior frontal triangularus	1.3	3668
Vermis 4 5	1.28	1266
Right postcentral gyrus	1.26	6549
Right cerebellum crus 2	1.25	3797
Left rolandic operculum	1.24	2187
Left cerebellum 7b	1.21	781
Left inferior temporal gyrus	1.18	6955
Right thalamus	1.16	1697
**OSB vs. HC, Class 1: OSB, *n* = 106, Class 2: HC, *n* = 27**		
Total accuracy, %	77	—
Balanced accuracy, %	63	*p* = 0.02
Class 1 predictive value, %	85	—
Class 2 predictive value, %	44	—
AUC	0.61	*p* = 0.01
Precision class 1/class 2	0.84/0.42	—
Recall class 1/class 2	0.87/0.37	—
**Region**	**Weights %**	**Voxels**
Right superior parietal gyrus	1.98	2733
Left cerebellum 10	1.75	117
Right cuneus	1.5	3089
Right cerebellum crus 2	1.49	3797
Left paracentral lobule	1.48	1905
Left superior parietal gyrus	1.45	3295
Right paracentral lobule	1.4	1457
Right superior inferior gyrus	1.38	6892
Left cuneus	1.36	3215
Left rolandic operculum	1.31	2187
Left inferior frontal operculum	1.29	1991
Left superior occipital gyrus	1.21	2436
Left inferior frontal triangularus	1.19	4360
Right angular	1.18	3407
Right postcentral gyrus	1.17	6549

Abbreviations: AUC = receiver operator characteristic area under curve; BD = bipolar disorder group; HC = healthy controls; OP = other psychopathology group; OSB = other specified bipolar disorder group.

### 2.6 Region‐Specific Classification Power and Linking to Clinical Symptoms

To explore which GMVs contributed most to classification, we used the AAL atlas (Tzourio‐Mazoyer et al. 2002) to compute SVM weight vectors for 116 cortical and subcortical anatomical structures (Schrouff et al. 2013; Schrouff et al. 2018; Haufe et al. 2014). We then examined the top 15 GMVs contributing most to discrimination. The linear model weight maps were interpreted as indicating the most discriminative voxels/ROIs in the high‐dimensional GM space, allowing us to localize these discriminative features to specific brain regions (Haufe et al. 2014).

Spearman correlation analysis of the top GMVs contributing to clinical classification models performance (BD vs. OSB and BD vs. OP) and clinical symptoms was performed in SPSS. We matched volumes based on Freesurfer's parcellation using the Destrieux atlas, aligning them with labels from the AAL atlas as implemented in PRoNTo. Significance was set at permutation‐based *p* < 0.05.

### 2.7 Sensitivity Analysis on Class Imbalance

For the BD versus OSB model and OSB versus HC models, which had the largest imbalance, we conducted additional sensitivity analyses using random undersampling within PRoNTo, we balanced the two classes at the smallest N subjects per group for each model and re‐ran the classification using the same CV procedure.

## 3 Results

### 3.1 Classification

#### 3.1.1 BD Versus OSB

In the classification model BD‐I/II versus OSB, using whole‐brain GMV, the total accuracy was 79%, with a predictive value for Class 1 (BD) of 67% and Class 2 (OSB) of 82%. All statistics for the classification model, including the area under curve (AUC = 0.70, *p* = 0.002), alongside balanced accuracy, precision, and recall are presented in Table 2. The 15 GMVs most informative for discrimination between BD and OSB were: in the right hemisphere: precentral gyrus, postcentral gyrus, superior parietal gyrus (SPG), caudate, cerebellum 7b, 8, 10; in the left hemisphere: paracentral lobule, temporal transverse (Heschl's) gyrus, SPG, angular gyrus, caudate, putamen, cerebellum 9, 10 (for full results see Table 2).

#### 3.1.2 BD‐I/II Versus OP

In the classification model BD‐I/II versus OP, the total accuracy was 66%, with a predictive value for Class 1 (BD) of 54% and Class 2 (OP) of 75%, AUC = 0.61, *p* = 0.014. The top 15 GMVs were: in the right hemisphere: frontal inferior operculum (IFO), paracentral lobule, SPG, caudate, cerebellum 3, 7b, 8, 10; in the left hemisphere: paracentral lobule, temporal transverse (Heschl's) gyrus, middle temporal pole, pallidum, cerebellum 7b, 9, 10 (for full results see Table 2).

#### 3.1.3 BD‐I/II Versus HC

In the classification model BD‐I/II versus HC, the total accuracy was 66%, with a predictive value for Class 1 (BD) of 69% and Class 2 (HC) of 67%, AUC = 0.67, *p* = 0.011. The top 15 GMVs were: in the right hemisphere: inferior parietal gyrus, middle occipital gyrus, cerebellum 10; in the left hemisphere: IFO, middle frontal gyrus, inferior frontal triangularis, supplemental motor area, paracentral lobule, SPG, angular gyrus, pallidum, cerebellum 9, 10, and Crus1; vermis 3 (for full results see Table 2).

#### 3.1.4 OP Versus HC

In the classification model OP versus HC, the total accuracy was 68%, with a predictive value for Class 1 (OP) of 75% and Class 2 (HC) of 53%, AUC = 0.70, *p* = 0.001. The top 15 GMVs were: in the right hemisphere: inferior frontal triangularis, precentral gyrus, paracentral lobule, postcentral gyrus, thalamus, cerebellum Crus2; in the left hemisphere: IFO, inferior temporal gyrus, middle temporal pole, Rolandic operculum, paracentral lobule, precuneus, cerebellum 7b, vermis 3, 4, 5 (for full results see Table 2).

#### 3.1.5 OSB Versus HC

In the classification model OSB versus HC, the total accuracy was 77%, with a predictive value for Class 1 (OSB of 85% and Class 2 (HC) of 44%, AUC = 0.61, *p* = 0.01. The top 15 GMVs were: in the right hemisphere: superior frontal gyrus, paracentral lobule, inferior parietal gyrus, SPG, cuneus, angular gyrus, cerebellum 7b, and Crus2; in the left hemisphere: IFO, Rolandic operculum, paracentral lobule, SPG, cuneus, cerebellum 10; Vermis 3 (for full results see Table 2).

### 3.2 Spearman Correlation Analysis

Right SPG volume was positively associated with negative affect in BD (rho = 0.37, *p* = 0.04) while in OSB bilateral SPG volume was negatively associated with negative affect (right SPG: rho = −0.39, *p* ≤ 0.001; left SPG: rho = −0.37, *p* = 0.04) and negatively associated with anxiety (rho = −0.26, *p* = 0.03). In the OSB group, bilateral cerebellar volumes were also negatively associated with negative affect (right cerebellum: rho = −0.3, *p* = 0.01; left cerebellum: rho = −0.29, *p* = 0.02), while the left transverse temporal gyrus was positively associated with self‐reported mania, both current (rho = 0.33, *p* = 0.01) and lifetime (rho = 0.31, *p* = 0.02). Left pallidum was positively related to current mania in BD (rho = 0.41, *p* = 0.04). In the OP group, the left paracentral cortical volume (rho = −0.32, *p* = 0.04), the right IFO (rho = −0.43, *p* = 0.01), and the right caudate (rho = −0.35, *p* = 0.02) were negatively related to self‐reported positive affect. No other significant relationships were observed (all *ps *> 0.06) (for full results see Table 3).

**TABLE 3:** Spearman correlations between identified gray matter volumes (GMVs) and self‐reported clinical measures of mania (BCMRS), depression (MFQ), anxiety (SCARED), and affect (PANAS). Statistically significant relationships are reported in bold and highlighted.

		BCMRS last month		BCMRS lifetime		MFQ		SCARED		PANAS positive		PANAS negative	
**BD‐I/II**	hemisphere	Observed Spearman correlation	Permutation‐based *p*‐value	Observed Spearman correlation	Permutation‐based *p*‐value	Observed Spearman correlation	Permutation‐based *p*‐value	Observed Spearman correlation	Permutation‐based *p*‐value	Observed Spearman correlation	Permutation‐based *p*‐value	Observed Spearman correlation	Permutation‐based *p*‐value
Cerebellum cortex	left	−0.06	0.77	−0.08	0.68	−0.18	0.33	−0.11	0.58	0.07	0.72	−0.08	0.67
Cerebellum cortex	right	−0.02	0.93	−0.05	0.81	−0.12	0.51	−0.04	0.83	0.16	0.42	−0.03	0.86
Transverse temporal (Heschl's) gyrus	left	0.03	0.89	−0.03	0.91	0.12	0.53	0.26	0.16	−0.07	0.7	0.2	0.3
Paracentral lobule	left	0.16	0.41	0.27	0.16	0.06	0.75	−0.03	0.87	0.12	0.54	0	1
Paracentral lobule	right	0.29	0.14	0.27	0.15	0.17	0.38	0.24	0.2	−0.12	0.5	0.25	0.17
Caudate	left	0.3	0.14	0.12	0.53	0.13	0.5	−0.25	0.18	0.24	0.18	0.03	0.86
Caudate	right	0.3	0.14	0.16	0.43	0.19	0.35	−0.13	0.5	0.12	0.53	0.08	0.69
Superior parietal gyrus	left	0.02	0.93	0.01	0.98	0.03	0.88	−0.06	0.76	0.01	0.97	0.23	0.22
Superior parietal gyrus	right	0.18	0.41	0.15	0.46	0.23	0.2	0.01	0.95	0.17	0.37	**0.37**	**0.04**
Pallidum	left	**0.41**	**0.04**	0.34	0.07	0.19	0.32	0.08	0.66	0.04	0.86	0.13	0.52
Inferior frontal operculum	right	0.23	0.25	0.29	0.14	0.09	0.62	−0.31	0.1	0.12	0.58	0.09	0.64
Temporal pole	left	−0.04	0.81	−0.25	0.22	−0.06	0.72	0.01	0.97	0	0.99	0.05	0.77
Angular gyrus	left	0.24	0.24	0.33	0.08	0.07	0.7	−0.11	0.58	0.13	0.51	0.26	0.16
Precentral gyrus	right	0.38	0.06	0.28	0.16	0.13	0.51	0.16	0.4	−0.09	0.66	0.34	0.07
Postcentral gyrus	right	0.19	0.32	0.26	0.18	0.23	0.22	0.1	0.57	−0.08	0.68	0.23	0.21
Putamen	left	0.21	0.3	0.14	0.48	0.02	0.9	−0.11	0.57	−0.16	0.41	0.02	0.93
**OSB**													
Cerebellum cortex	left	−0.01	0.93	0.08	0.54	−0.1	0.41	−0.24	0.05	0.22	0.07	**−0.29**	**0.02**
Cerebellum cortex	right	0.06	0.7	0.15	0.25	−0.1	0.42	−0.18	0.14	0.25	0.05	**−0.3**	**0.01**
Transverse temporal (Heschl's) gyrus	left	**0.33**	**0.01**	**0.31**	**0.02**	−0.04	0.71	−0.1	0.45	0.11	0.39	−0.15	0.23
Paracentral lobule	left	0.02	0.86	−0.05	0.7	−0.04	0.76	−0.13	0.3	−0.05	0.64	−0.09	0.48
Caudate	left	−0.04	0.74	0.12	0.37	0.03	0.84	−0.05	0.73	0.01	0.92	−0.12	0.34
Caudate	right	0.03	0.85	0.19	0.13	−0.01	0.93	−0.06	0.64	0.08	0.49	−0.14	0.27
Superior parietal gyrus	left	−0.09	0.47	−0.03	0.81	−0.17	0.15	−0.21	0.09	−0.04	0.77	**−0.31**	**0.01**
Superior parietal gyrus	right	−0.03	0.86	−0.05	0.69	−0.14	0.23	**−0.26**	**0.03**	−0.01	0.94	**−0.39**	**<.001**
Angular gyrus	left	−0.05	0.68	0	0.99	−0.07	0.55	−0.02	0.86	0.01	0.91	−0.11	0.34
Precentral gyrus	right	0.23	0.05	0.11	0.38	−0.01	0.94	−0.18	0.15	0.09	0.48	−0.12	0.3
Postcentral gyrus	right	0.07	0.62	0.06	0.67	0.13	0.28	0.03	0.83	0.03	0.79	−0.08	0.45
Putamen	left	−0.04	0.79	−0.06	0.68	−0.01	0.96	−0.09	0.46	0.02	0.85	−0.12	0.33
**OP**													
Cerebellum cortex	left	0.09	0.59	0.18	0.29	−0.1	0.52	−0.07	0.67	−0.22	0.17	−0.08	0.59
Cerebellum cortex	right	0.05	0.76	0.14	0.4	−0.06	0.7	−0.05	0.77	−0.2	0.22	−0.03	0.87
Transverse temporal (Heschl's) gyrus	left	−0.03	0.85	0.09	0.6	−0.13	0.42	0.04	0.79	−0.07	0.68	0.11	0.49
Paracentral lobule	left	−0.22	0.18	−0.08	0.61	−0.12	0.47	−0.06	0.73	**−0.32**	**0.04**	0	1
Paracentral lobule	right	−0.08	0.62	−0.12	0.45	−0.02	0.89	0.01	0.94	−0.06	0.7	0.07	0.67
Caudate	right	−0.22	0.16	−0.06	0.71	−0.16	0.35	−0.02	0.9	**−0.35**	**0.02**	0.06	0.66
Superior parietal gyrus	right	0.01	0.94	0.04	0.81	−0.07	0.66	−0.03	0.84	−0.23	0.13	0.12	0.44
Pallidum	left	−0.18	0.27	−0.14	0.37	−0.12	0.47	−0.05	0.77	−0.26	0.1	−0.02	0.93
Inferior frontal operculum	right	0.02	0.89	0	0.99	0.29	0.06	0.28	0.07	**−0.43**	**0.01**	0.19	0.21
Temporal pole	left	0.09	0.56	−0.09	0.61	0.18	0.28	0.24	0.12	−0.1	0.51	0.06	0.73

**Abbreviations**: BCMRS = Brief Child Mania Rating Scale; BD = bipolar disorder; MFQ = Mood and Feelings Questionnaire; OP = other psychopathology; OSB = other specified bipolar disorder; PANAS = Positive and Negative Affect Schedule; SCARED = Screen for Child Anxiety Related Disorders.

### 3.3 Sensitivity Analysis: Class Imbalance

After random undersampling in PRoNTo to balance BD and OSB at *n* = 34 subjects per group, the classification model achieved a total accuracy of 64.77% (AUC = 0.72, *p* = 0.004), and balanced accuracy of 64.77% (*p* = 0.034). Class predictive values were 67.58% (BD) and 69.24% (OSB). For BD the precision was 0.66 and the recall was 0.62, for OSB the precision was 0.64 and the recall was 0.68. After random undersampling to balance OSB and HC at *n* = 27 subjects per group, the classification model achieved a total accuracy of 69.44% (AUC = 0.7, *p* = 0.001), and balanced accuracy also 69.44% (*p* = 0.01). Class predictive values were 68.75% (OSB) and 76.39% (HC). For OSB the precision was 0.68 and the recall was 0.78, for HC the precision was 0.74 and the recall was 0.63.

## 4 Discussion

The present study used a whole‐brain machine learning SVM to classify the unique cohort of well‐characterized, severely ill adolescent inpatients with BD‐I/II, other specified bipolar disorder (OSB), and other psychopathology (OP), (as well as HC), based on whole‐brain GM volumes. Our first hypothesis was partially confirmed: the classification accuracies (66%–79%) significantly exceeded chance levels, particularly for BD versus OSB (79%), suggesting that GMVs, especially in areas associated with movement, balance, cognition, and sensory processing, can aid in differentiating BD‐I/II from both OSB and OP. Models performances were consistent after adjusting for class size imbalances. Our second hypothesis was also supported, as we observed significant correlations between top‐weighted GM regions and clinical symptoms of mania, anxiety, or affect.

Interestingly, classification accuracy was highest (79%) for distinguishing BD‐I/II from OSB, a finding that initially appears counterintuitive given the increased risk of conversion to BD‐I/II in OSB and the observed similarities between mania/hypomania and subthreshold mania/hypomania symptoms between these two groups. Several possibilities, both clinical and methodological may account for this: (1) Given that only a subgroup of OSB individuals truly show subthreshold mania/hypomania that is prodromal to BD, this group may have more heterogeneous neuroanatomic GMV patterns, reflecting their subthreshold mania/hypomania presentation and varied risk for developing either BD or OP; (2) a subgroup of OSB may represent an early stage or more “mixed” presentation with distinct neural alterations (Petruso et al. 2023). (3) The imbalanced class structure of our data may calculate overly optimistic classification results as suggested by the precision‐recall curve for this comparison (Figure 1). Our sensitivity analysis however suggests consistent performance after adjusting class size imbalance. Further research examining longitudinal changes in GM volumes with symptom development and studies with larger samples could clarify these patterns.

The identified regions included both cortical and subcortical areas of bilateral cerebellar volumes, paracentral lobules, striatal structures (e.g., caudate and pallidum), frontal opercular regions, and superior parietal gyri. While these regions are anatomically distributed, each region converges on aspects of movement, sensory processing, and/or cognitive control—domains increasingly recognized as potentially transdiagnostic (Keramatian et al. 2021; Colombo et al. 2022; Brown et al. 2020; Frank van den Boogert et al. 2022).

Specifically, across our diagnostic group classification models, bilateral cerebellar volumes functionally associated with motor control and learning, cognitive, and affective functions  (Roostaei et al. 2014; Van Essen et al. 2018), and bilateral paracentral lobule volume associated with motor and sensory function (Nachev et al. 2008) were identified as key GMVs when differentiating BD‐I/II. The associations of these GMVs with movement abnormalities are reflected in clinical characteristics of mania (motor hyperactivity, increased activity/speech) and depression (decreased overall motor activity, slower motor responses) symptomatology (Regier et al. 2013) and balance and gait abnormalities observed in mood disorders (Kang et al. 2019; Kang et al. 2018). These GMVs have been shown to differentiate BD‐I/II from healthy youth (Saxena et al. 2023; H. Zhang et al. 2022), however, to our knowledge, no studies have considered the classification power of neural regions related to movement in mood disorders. In addition, other movement‐related GMVs were identified in our other classification models such as caudate, putamen, cerebellum vermis, and supplemental motor area. These stable GM structures related to movement and balance and their clinical characteristics may be an important component of diagnostic classification that has not been fully considered in psychiatric research and diagnostic classification. Interestingly, the cerebellar function was shown to differentiate healthy adults from adults with subtypes of pediatric‐onset BD from the Course and Outcome of Bipolar Youth study (COBY) who had been clinically followed for up to 17.5 years prior to a neuroimaging assessment (Bertocci et al. 2020). These results may play an important role in motor function in BD versus other disorders that may be used in risk calculation and need further study.

Sensory processing abnormalities have recently been identified as a potential cross‐diagnostic marker of psychopathology (Brown et al. 2020; Frank van den Boogert et al. 2022) and two GMVs classifying BD in our models, SPG volume and inferior frontal operculum (IFO) volume, are associated with these clinical abnormalities in animals and humans (Dziedzic et al. 2021; Seghier 2013; Yoshimura et al. 2017). Lesion studies have shown that the superior parietal cortex is involved in generating and maintaining sensory and motor representations (Berlucchi and Aglioti 1997; Sirigu et al. 1996; Wolpert et al. 1998) and abnormal SPG volumes have been reported in mood disorders (Keramatian et al. 2021; Zheng et al. 2021; Kilic et al. 2024). We also show different diagnostic‐related relationships between SPG and symptom measures. Specifically, BD showed positive relationships between SPG volume and negative affect and OSB showed negative relationships between SPG volume and both negative affect and anxiety. These results support the potential role of SPG GMV abnormalities in contributing to risk calculation.

IFO is also involved in sensory sensitivity (Yoshimura et al. 2017), speech, and cognition and plays a key role in facial emotion expression processing (van der Gaag et al. 2007; Jabbi and Keysers 2008), subcomponents of sensory processing. We show right IFO volume as an important feature in the BD versus OP model while left IFO was identified in BD versus HC. This may be in line with a neural modulation study showing right IFO stimulation enhanced discrimination of faces expressing anger (Iarrobino et al. 2021), a high arousal emotion. Deficits in discriminating high arousal emotions are often observed in disruptive behavior disorders (Fernandez and Johnson 2016; Harty et al. 2009; Turgay 2004) (OP group) and we further show that IFO volumes in the OP group are negatively related to self‐reported positive affect. While GMVs in SPG and IFO were observed as important markers across all BD classification models, volumes in other sensory processing regions of the postcentral gyrus, thalamus, Rolandic operculum, and temporal lobe, were identified in our other classification models, supporting the role of neural correlates of sensory processing abnormalities in future risk classification specifically for BD.

Pallidum volume was also identified as an important classification feature in our BD models. It is involved in planning and implementing goal‐oriented behavior (Ottenheimer et al. 2020) and has been shown to differ between BD and healthy controls (Sandoval et al. 2016; Strakowski et al. 1999). Left pallidum volume was positively related to self‐reported mania in the past month in BD but not in the OP group. GMVs of other regions involved in learning such as caudate, putamen, and angular gyrus were also identified but were not associated with our clinical self‐report measures. Future studies can examine relationships with other clinical measures.

This study had some limitations. While the unique inpatient sample is a strength, and data loss was relatively low, a larger sample study would likely improve classifier performance and generalizability. Although 66% accuracy may be below the threshold for direct clinical utility, it surpasses chance and underscores the potential for neuroimaging‐informed diagnostic tools (Mateos‐Pérez et al. 2018; Winter et al. 2024). Notably, 79% accuracy for BD‐I/II versus OSB classification is encouraging, considering the clinical difficulty of differentiating full manic episode presentations from subthreshold mania/hypomania symptoms which may or may not truly be prodromal to BD. The sensitivity analysis has shown that the predictive value for BD was similar after balancing sample sizes with an undersampling approach, suggesting that this metric was not artificially inflated by the original class imbalance. However, the higher total accuracy in our original model was likely partially influenced by class imbalance (Thölke et al. 2023; Buda et al. 2018), underscoring the importance of balanced or weighted approaches in future studies. In addition, there were differences in age and sex among the inpatient participants. This likely reflects the known disparities in help‐seeking between females and males. The morphometry even across healthy individuals has been shown to differ (Tomaiuolo et al. 1999) and our findings using standard computer‐generated parcellation methods may overlook differences in brain morphometry. Yet, we show good accuracy in differentiating diagnostic groups. Due to the cross‐sectional design of this study, our analyses cannot determine whether observed GM patterns are risk markers or reflect the effects of illness burden and/or treatment. Longitudinal follow‐up is needed. Findings regarding differences between BD‐I/II and OSB underscore that some individuals with subthreshold mania/hypomania symptoms exhibit GMVs that are distinct from those with full‐threshold BD‐I/II. Since OSB is at elevated risk for progression to BD‐I/II, future longitudinal studies could test whether GM patterns at baseline predict transition to BD. Severe forms of psychopathology that were present in our sample might improve our identifying biological markers of BD, but the findings may not be generalizable to less severe community cases with BD; thus, our findings would need to be replicated in larger community samples. Finally, since our priority in this study was the interpretability of voxel‐level weights and minimizing overfitting in a relatively moderate sample size we used a linear SVM approach, but future research may benefit from employing other machine learning techniques such as Random Forest, gradient boosting, deep learning methods, and multimodal data integration (e.g., structural, functional, diffusion MRI).

In summary, this study demonstrates the potential of using a whole‐brain linear SVM classifier of GMVs to detect biomarkers of BD in adolescents. We show that classification can successfully distinguish not only BD from healthy controls but also BD‐I/II from OSB and OP. The implicated structural areas, particularly those involved in movement, sensory processing, and cognitive control, should be investigated in future longitudinal or multimodal imaging designs to better understand their role in the pathophysiology and differential diagnosis of pediatric mood disorders. These findings may inform future studies aimed at developing a diagnostic risk calculator of psychopathology to facilitate earlier and more accurate diagnostic processes.

## Author Contributions

**Renata Rozovsky**: conceptualization, writing–original draft, methodology, validation, visualization, writing–review and editing, data curation, formal analysis. **Maria Wolfe**: writing–review and editing, supervision, data curation, project administration, investigation. **Halimah Abdul–waalee**: investigation, writing–review and editing. **Mariah Chobany**: writing–review and editing, investigation. **Greeshma Malgireddy**: writing–review and editing, data curation. **Jonathan A. Hart**: data curation, writing–review and editing. **Brianna Lepore**: writing–review and editing, investigation. **Farzan Vahedifard**: writing–review and editing. **Mary L. Phillips**: writing–review and editing. **Boris Birmaher**: writing–review and editing. **Alex Skeba**: software, writing–review and editing, resources, data curation. **Rasim S. Diler**: conceptualization, writing–review and editing, funding acquisition. **Michele A. Bertocci**: funding acquisition, conceptualization, methodology, visualization, writing–original draft, writing–review and editing.

## Ethics Statement

The authors assert that all procedures contributing to this work comply with the ethical standards of the relevant national and institutional committees on human experimentation and with the Helsinki Declaration of 1975, as revised in 2008.

## Conflicts of Interest

The authors declare no conflicts of interest.

## Peer Review

The peer review history for this article is available at https://publons.com/publon/10.1002/brb3.70589

## Data Availability

The data that support the findings of this study are available on request from the corresponding author. The data are not publicly available due to privacy or ethical restrictions.
